# 1-(2,3,4-Trihydroxy­benzyl­idene)thio­semicarbazide

**DOI:** 10.1107/S1600536810015850

**Published:** 2010-05-08

**Authors:** Hana Bashir Shawish, M. Jamil Maah, Seik Weng Ng

**Affiliations:** a Department of Chemistry, University of Malaya, 50603 Kuala Lumpur, Malaysia

## Abstract

In the title mol­ecule, C_8_H_9_N_3_O_3_S, the thio­semicarbazide =N—NH—C(=S)—NH— fragment is twist a different degree of twist in the three independent mol­ecules [dihedral angles = 7.6 (1), 11.6 (1) and 20.7 (1)°]. Intra­molecular O—H⋯N and O—H⋯O hydrogen bonds occur. In the crystal, the hydr­oxy and amino groups are hydrogen-bond donors and the O—H⋯O, O—H⋯S and N—H⋯O hydrogen bonds generate a layer motif.

## Related literature

For the crystal structures of 2,4-dihydroxy­benzaldehyde thio­semicarbazone and 3,4-dihydroxy­benzaldehyde thio­semi­carba­zone, see: Swesi *et al.* (2006 ▶); Tan *et al.* (2008 ▶).
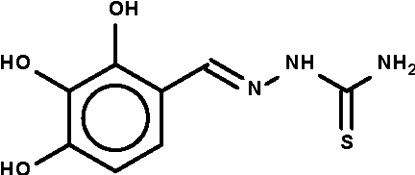


## Experimental

### 

#### Crystal data

C_8_H_9_N_3_O_3_S
*M* *_r_* = 227.24
Triclinic, 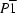

*a* = 10.3121 (10) Å
*b* = 11.8797 (12) Å
*c* = 12.4037 (12) Å
α = 68.969 (1)°
β = 87.487 (1)°
γ = 77.161 (1)°
*V* = 1381.8 (2) Å^3^
*Z* = 6
Mo *K*α radiation
μ = 0.34 mm^−1^
*T* = 100 K
0.35 × 0.10 × 0.02 mm

#### Data collection

Bruker SMART APEX diffractometer
Absorption correction: multi-scan (*SADABS*; Sheldrick, 1996 ▶) *T*_min_ = 0.890, *T*_max_ = 0.993
13248 measured reflections
6327 independent reflections
4487 reflections with *I* > 2σ(*I*)
*R*_int_ = 0.033

#### Refinement

*R*[*F* ^2^ > 2σ(*F* ^2^)] = 0.042
*wR*(*F* ^2^) = 0.114
*S* = 1.02
6327 reflections
478 parameters
18 restraints
H atoms treated by a mixture of independent and constrained refinement
Δρ_max_ = 0.38 e Å^−3^
Δρ_min_ = −0.37 e Å^−3^

### 

Data collection: *APEX2* (Bruker, 2009 ▶); cell refinement: *SAINT* (Bruker, 2009 ▶); data reduction: *SAINT*; program(s) used to solve structure: *SHELXS97* (Sheldrick, 2008 ▶); program(s) used to refine structure: *SHELXL97* (Sheldrick, 2008 ▶); molecular graphics: *X-SEED* (Barbour, 2001 ▶); software used to prepare material for publication: *publCIF* (Westrip, 2010 ▶).

## Supplementary Material

Crystal structure: contains datablocks global, I. DOI: 10.1107/S1600536810015850/pk2242sup1.cif

Structure factors: contains datablocks I. DOI: 10.1107/S1600536810015850/pk2242Isup2.hkl

Additional supplementary materials:  crystallographic information; 3D view; checkCIF report

## Figures and Tables

**Table 1:** Hydrogen-bond geometry (Å, °)

*D*—H⋯*A*	*D*—H	H⋯*A*	*D*⋯*A*	*D*—H⋯*A*
O1—H1⋯N1	0.84 (1)	1.94 (2)	2.682 (2)	147 (3)
O2—H2⋯O3^i^	0.83 (1)	2.17 (2)	2.773 (2)	129 (3)
O3—H3⋯S1^ii^	0.83 (1)	2.36 (1)	3.184 (2)	173 (3)
O4—H4⋯N4	0.84 (1)	1.96 (2)	2.666 (2)	141 (3)
O5—H5⋯O6	0.83 (1)	2.24 (3)	2.730 (2)	118 (3)
O6—H6⋯S2^ii^	0.84 (1)	2.45 (1)	3.276 (2)	172 (2)
O7—H7⋯N7	0.83 (1)	1.97 (2)	2.710 (2)	148 (3)
O8—H8⋯O9	0.84 (1)	2.24 (3)	2.697 (2)	114 (2)
O9—H9⋯S3^iii^	0.84 (1)	2.40 (1)	3.237 (2)	174 (2)
N3—H31⋯O2^iv^	0.85 (1)	2.09 (1)	2.902 (2)	159 (3)
N6—H61⋯O8^v^	0.86 (1)	2.01 (1)	2.847 (2)	164 (2)
N9—H91⋯O5^vi^	0.86 (1)	2.11 (2)	2.896 (2)	153 (2)

Symmetry codes: (i) 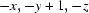; (ii) 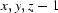; (iii) 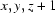; (iv) 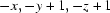; (v) 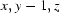; (vi) 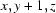.

**Fig. 1.**
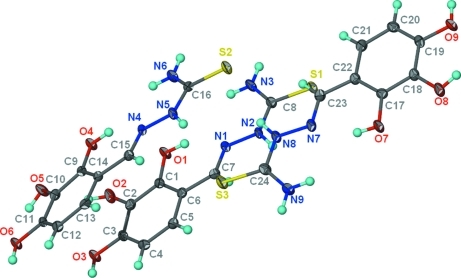
Thermal ellipsoid plot (Barbour, 2001) of the three independent molecules of C8H9N3O3S at the 70% probability level; hydrogen atoms are drawn as spheres of arbitrary radius.

## supplementary crystallographic information

### Experimental

2,3,4-Trihydroxybenzaldehyde (1.54 g, 10 mmol) and thiosemicarbazide (0.91 g, 1 mmol) were heated in ethanol (20 ml). The cool solution was set aside for the growth of crystals.

### Refinement

Carbon-bound H-atoms were placed in calculated positions (C—H 0.95 to 0.98 Å) and were included in the refinement in the riding model approximation, with *U*(H) set to 1.2 or 1.5*U*(C_Me_).

The amino and hydroxy H-atoms were located in a difference Fourier map, and were refined with distance restraints of N–H 0.86 (1) and O–H 0.84 (1) Å; their temperature factors were freely refined.

### Crystal data

**Table:**

C_8_H_9_N_3_O_3_S	*Z* = 6
*M**_r_* = 227.24	*F*(000) = 708
Triclinic, *P*1	*D*_x_ = 1.639 Mg m^−^^3^
Hall symbol: -P 1	Mo *K*α radiation, λ = 0.71073 Å
*a* = 10.3121 (10) Å	Cell parameters from 2924 reflections
*b* = 11.8797 (12) Å	θ = 2.6–28.1°
*c* = 12.4037 (12) Å	µ = 0.34 mm^−^^1^
α = 68.969 (1)°	*T* = 100 K
β = 87.487 (1)°	Plate, pale brown
γ = 77.161 (1)°	0.35 × 0.10 × 0.02 mm
*V* = 1381.8 (2) Å^3^	

### Data collection

**Table:**

Bruker SMART APEX diffractometer	6327 independent reflections
Radiation source: fine-focus sealed tube	4487 reflections with *I* > 2σ(*I*)
graphite	*R*_int_ = 0.033
ω scans	θ_max_ = 27.5°, θ_min_ = 1.8°
Absorption correction: multi-scan (*SADABS*; Sheldrick, 1996)	*h* = −13→12
*T*_min_ = 0.890, *T*_max_ = 0.993	*k* = −15→15
13248 measured reflections	*l* = −16→16

### Refinement

**Table:**

Refinement on *F*^2^	Primary atom site location: structure-invariant direct methods
Least-squares matrix: full	Secondary atom site location: difference Fourier map
*R*[*F*^2^ > 2σ(*F*^2^)] = 0.042	Hydrogen site location: inferred from neighbouring sites
*wR*(*F*^2^) = 0.114	H atoms treated by a mixture of independent and constrained refinement
*S* = 1.02	*w* = 1/[σ^2^(*F*_o_^2^) + (0.0572*P*)^2^ + 0.0368*P*] where *P* = (*F*_o_^2^ + 2*F*_c_^2^)/3
6327 reflections	(Δ/σ)_max_ < 0.001
478 parameters	Δρ_max_ = 0.38 e Å^−^^3^
18 restraints	Δρ_min_ = −0.37 e Å^−^^3^

### Fractional atomic coordinates and isotropic or equivalent isotropic displacement parameters (Å^2^)

**Table:**

	*x*	*y*	*z*	*U*_iso_*/*U*_eq_	
S1	0.04483 (6)	0.85739 (5)	0.67520 (4)	0.01600 (14)	
S2	0.39952 (6)	0.72567 (5)	0.55744 (5)	0.01715 (14)	
S3	0.32855 (6)	1.02438 (5)	0.19484 (5)	0.01889 (15)	
O1	0.02467 (17)	0.58729 (14)	0.32793 (12)	0.0171 (4)	
H1	0.030 (3)	0.622 (2)	0.3749 (19)	0.033 (8)*	
O2	−0.01767 (17)	0.52286 (14)	0.14650 (13)	0.0185 (4)	
H2	0.023 (3)	0.489 (3)	0.103 (2)	0.048 (10)*	
O3	0.00587 (16)	0.67307 (14)	−0.07208 (12)	0.0165 (3)	
H3	0.019 (3)	0.725 (2)	−0.1352 (14)	0.031 (8)*	
O4	0.30904 (16)	0.48727 (14)	0.20588 (12)	0.0159 (3)	
H4	0.325 (3)	0.514 (3)	0.257 (2)	0.054 (10)*	
O5	0.23954 (17)	0.43594 (14)	0.02850 (13)	0.0180 (4)	
H5	0.218 (3)	0.438 (3)	−0.0364 (14)	0.063 (11)*	
O6	0.30786 (16)	0.55735 (14)	−0.19098 (13)	0.0166 (3)	
H6	0.326 (3)	0.607 (2)	−0.2539 (13)	0.030 (8)*	
O7	0.27294 (17)	1.30858 (14)	0.54062 (13)	0.0184 (4)	
H7	0.287 (3)	1.270 (2)	0.496 (2)	0.038 (9)*	
O8	0.27623 (17)	1.38726 (14)	0.71552 (13)	0.0182 (4)	
H8	0.284 (3)	1.399 (3)	0.7771 (16)	0.051 (10)*	
O9	0.28539 (16)	1.23273 (14)	0.93746 (12)	0.0168 (3)	
H9	0.294 (3)	1.1754 (17)	1.0024 (12)	0.025 (7)*	
N1	0.05073 (18)	0.77060 (16)	0.39755 (14)	0.0124 (4)	
N2	0.05168 (19)	0.83290 (16)	0.47275 (15)	0.0131 (4)	
H21	0.045 (2)	0.9116 (10)	0.445 (2)	0.024 (7)*	
N3	0.0408 (2)	0.65715 (17)	0.62610 (16)	0.0194 (4)	
H31	0.033 (3)	0.620 (2)	0.6981 (10)	0.036 (8)*	
H32	0.038 (2)	0.6192 (19)	0.5797 (17)	0.018 (7)*	
N4	0.39153 (18)	0.64797 (15)	0.27613 (15)	0.0126 (4)	
N5	0.40933 (19)	0.70383 (17)	0.35334 (15)	0.0135 (4)	
H51	0.425 (3)	0.7772 (14)	0.324 (2)	0.045 (9)*	
N6	0.3389 (2)	0.54900 (17)	0.49721 (16)	0.0177 (4)	
H61	0.325 (2)	0.510 (2)	0.5681 (10)	0.020 (7)*	
H62	0.334 (2)	0.515 (2)	0.4482 (16)	0.019 (7)*	
N7	0.30054 (19)	1.11133 (16)	0.47366 (15)	0.0153 (4)	
N8	0.3167 (2)	1.04604 (17)	0.39915 (15)	0.0163 (4)	
H81	0.337 (2)	0.9667 (9)	0.425 (2)	0.019 (7)*	
N9	0.2893 (2)	1.22846 (17)	0.24562 (16)	0.0200 (4)	
H91	0.284 (2)	1.270 (2)	0.1728 (9)	0.024 (7)*	
H92	0.278 (3)	1.265 (2)	0.2949 (18)	0.034 (8)*	
C1	0.0359 (2)	0.66793 (19)	0.21952 (17)	0.0121 (4)	
C2	0.0183 (2)	0.63384 (18)	0.12652 (18)	0.0129 (4)	
C3	0.0300 (2)	0.71265 (19)	0.01372 (17)	0.0123 (4)	
C4	0.0646 (2)	0.82510 (19)	−0.00648 (18)	0.0139 (5)	
H4A	0.0770	0.8772	−0.0829	0.017*	
C5	0.0806 (2)	0.85971 (19)	0.08633 (17)	0.0129 (4)	
H5A	0.1033	0.9368	0.0726	0.015*	
C6	0.0645 (2)	0.78427 (19)	0.19995 (17)	0.0117 (4)	
C7	0.0709 (2)	0.83158 (19)	0.29141 (17)	0.0123 (4)	
H7A	0.0908	0.9106	0.2726	0.015*	
C8	0.0451 (2)	0.77519 (19)	0.58732 (18)	0.0136 (5)	
C9	0.3408 (2)	0.55759 (19)	0.09885 (17)	0.0125 (4)	
C10	0.3076 (2)	0.52858 (18)	0.00573 (18)	0.0129 (4)	
C11	0.3422 (2)	0.59381 (19)	−0.10491 (17)	0.0120 (4)	
C12	0.4069 (2)	0.68949 (19)	−0.12458 (18)	0.0138 (5)	
H12	0.4322	0.7323	−0.2002	0.017*	
C13	0.4343 (2)	0.72205 (19)	−0.03270 (18)	0.0135 (4)	
H13	0.4767	0.7889	−0.0463	0.016*	
C14	0.4006 (2)	0.65829 (19)	0.07964 (17)	0.0119 (4)	
C15	0.4214 (2)	0.70176 (19)	0.17123 (18)	0.0126 (4)	
H15	0.4584	0.7725	0.1532	0.015*	
C16	0.3804 (2)	0.65349 (19)	0.46518 (18)	0.0130 (4)	
C17	0.2798 (2)	1.22282 (19)	0.64927 (18)	0.0135 (4)	
C18	0.2795 (2)	1.26454 (19)	0.74128 (18)	0.0134 (5)	
C19	0.2838 (2)	1.1822 (2)	0.85449 (18)	0.0134 (4)	
C20	0.2863 (2)	1.0579 (2)	0.87772 (18)	0.0152 (5)	
H20	0.2871	1.0021	0.9551	0.018*	
C21	0.2877 (2)	1.0171 (2)	0.78646 (18)	0.0153 (5)	
H21a	0.2898	0.9324	0.8020	0.018*	
C22	0.2860 (2)	1.09729 (19)	0.67167 (18)	0.0133 (4)	
C23	0.2966 (2)	1.0449 (2)	0.58130 (18)	0.0146 (5)	
H23	0.3008	0.9590	0.6023	0.018*	
C24	0.3104 (2)	1.1070 (2)	0.28385 (18)	0.0149 (5)	

### Atomic displacement parameters (Å^2^)

**Table:**

	*U*^11^	*U*^22^	*U*^33^	*U*^12^	*U*^13^	*U*^23^
S1	0.0281 (4)	0.0122 (3)	0.0102 (2)	−0.0063 (2)	0.0013 (2)	−0.0060 (2)
S2	0.0270 (4)	0.0164 (3)	0.0125 (3)	−0.0084 (2)	0.0028 (2)	−0.0086 (2)
S3	0.0312 (4)	0.0155 (3)	0.0128 (3)	−0.0074 (2)	0.0010 (2)	−0.0070 (2)
O1	0.0326 (10)	0.0128 (8)	0.0088 (7)	−0.0099 (7)	0.0009 (7)	−0.0042 (6)
O2	0.0340 (11)	0.0120 (8)	0.0157 (8)	−0.0124 (7)	0.0038 (7)	−0.0083 (6)
O3	0.0283 (10)	0.0156 (8)	0.0096 (7)	−0.0104 (7)	0.0011 (7)	−0.0059 (6)
O4	0.0279 (10)	0.0134 (8)	0.0093 (7)	−0.0097 (7)	0.0017 (6)	−0.0046 (6)
O5	0.0321 (10)	0.0160 (8)	0.0105 (7)	−0.0138 (7)	−0.0001 (7)	−0.0052 (6)
O6	0.0286 (10)	0.0147 (8)	0.0097 (7)	−0.0082 (7)	0.0000 (7)	−0.0061 (6)
O7	0.0345 (11)	0.0124 (8)	0.0108 (7)	−0.0075 (7)	0.0019 (7)	−0.0060 (6)
O8	0.0319 (10)	0.0132 (8)	0.0141 (8)	−0.0097 (7)	0.0035 (7)	−0.0079 (6)
O9	0.0257 (10)	0.0178 (8)	0.0099 (7)	−0.0080 (7)	0.0008 (7)	−0.0066 (6)
N1	0.0151 (10)	0.0125 (9)	0.0122 (9)	−0.0039 (7)	−0.0017 (7)	−0.0068 (7)
N2	0.0222 (11)	0.0088 (9)	0.0109 (8)	−0.0041 (8)	−0.0001 (7)	−0.0063 (7)
N3	0.0399 (13)	0.0113 (9)	0.0096 (9)	−0.0090 (9)	−0.0003 (9)	−0.0047 (7)
N4	0.0171 (10)	0.0107 (9)	0.0128 (9)	−0.0025 (7)	−0.0020 (7)	−0.0075 (7)
N5	0.0200 (11)	0.0111 (9)	0.0135 (9)	−0.0057 (8)	0.0003 (7)	−0.0079 (7)
N6	0.0321 (12)	0.0126 (9)	0.0107 (9)	−0.0083 (8)	0.0021 (8)	−0.0049 (8)
N7	0.0226 (11)	0.0114 (9)	0.0154 (9)	−0.0053 (8)	0.0004 (8)	−0.0082 (7)
N8	0.0303 (12)	0.0092 (9)	0.0117 (9)	−0.0059 (8)	0.0005 (8)	−0.0056 (7)
N9	0.0373 (13)	0.0114 (9)	0.0119 (9)	−0.0077 (9)	−0.0011 (9)	−0.0034 (8)
C1	0.0140 (12)	0.0109 (10)	0.0107 (10)	−0.0023 (8)	0.0013 (8)	−0.0035 (8)
C2	0.0151 (12)	0.0091 (10)	0.0156 (10)	−0.0040 (8)	0.0003 (9)	−0.0048 (8)
C3	0.0131 (12)	0.0138 (10)	0.0118 (10)	−0.0033 (9)	0.0002 (8)	−0.0065 (8)
C4	0.0166 (12)	0.0126 (10)	0.0109 (10)	−0.0040 (9)	0.0014 (8)	−0.0018 (8)
C5	0.0161 (12)	0.0087 (10)	0.0146 (10)	−0.0035 (8)	−0.0004 (8)	−0.0044 (8)
C6	0.0117 (11)	0.0104 (10)	0.0137 (10)	−0.0022 (8)	−0.0005 (8)	−0.0051 (8)
C7	0.0132 (12)	0.0102 (10)	0.0143 (10)	−0.0039 (8)	−0.0018 (8)	−0.0044 (8)
C8	0.0165 (12)	0.0136 (10)	0.0121 (10)	−0.0044 (9)	0.0006 (8)	−0.0056 (8)
C9	0.0150 (12)	0.0112 (10)	0.0104 (9)	−0.0009 (8)	0.0011 (8)	−0.0040 (8)
C10	0.0169 (12)	0.0077 (10)	0.0150 (10)	−0.0043 (9)	0.0004 (8)	−0.0042 (8)
C11	0.0161 (12)	0.0107 (10)	0.0109 (10)	−0.0014 (8)	−0.0020 (8)	−0.0064 (8)
C12	0.0165 (12)	0.0125 (10)	0.0124 (10)	−0.0039 (9)	0.0015 (8)	−0.0041 (8)
C13	0.0132 (12)	0.0111 (10)	0.0156 (10)	−0.0022 (8)	−0.0016 (8)	−0.0040 (8)
C14	0.0135 (12)	0.0105 (10)	0.0129 (10)	−0.0018 (8)	−0.0020 (8)	−0.0056 (8)
C15	0.0138 (12)	0.0108 (10)	0.0144 (10)	−0.0021 (8)	−0.0016 (8)	−0.0059 (8)
C16	0.0143 (12)	0.0113 (10)	0.0128 (10)	−0.0006 (9)	−0.0007 (8)	−0.0046 (8)
C17	0.0160 (12)	0.0123 (10)	0.0116 (10)	−0.0041 (9)	0.0001 (8)	−0.0031 (8)
C18	0.0163 (12)	0.0120 (10)	0.0148 (10)	−0.0057 (9)	0.0017 (9)	−0.0067 (8)
C19	0.0118 (12)	0.0181 (11)	0.0134 (10)	−0.0050 (9)	0.0033 (8)	−0.0085 (9)
C20	0.0179 (13)	0.0149 (11)	0.0112 (10)	−0.0045 (9)	0.0011 (9)	−0.0025 (8)
C21	0.0202 (13)	0.0102 (10)	0.0161 (11)	−0.0048 (9)	0.0007 (9)	−0.0044 (8)
C22	0.0148 (12)	0.0125 (10)	0.0145 (10)	−0.0045 (9)	0.0005 (8)	−0.0063 (8)
C23	0.0171 (12)	0.0123 (10)	0.0165 (11)	−0.0046 (9)	−0.0007 (9)	−0.0066 (9)
C24	0.0174 (12)	0.0155 (11)	0.0136 (10)	−0.0068 (9)	0.0002 (9)	−0.0052 (9)

### Geometric parameters (Å, °)

**Table:**

S1—C8	1.704 (2)	N7—N8	1.388 (2)
S2—C16	1.698 (2)	N8—C24	1.348 (3)
S3—C24	1.702 (2)	N8—H81	0.858 (10)
O1—C1	1.360 (2)	N9—C24	1.316 (3)
O1—H1	0.836 (10)	N9—H91	0.857 (10)
O2—C2	1.383 (2)	N9—H92	0.861 (10)
O2—H2	0.834 (10)	C1—C2	1.383 (3)
O3—C3	1.360 (2)	C1—C6	1.411 (3)
O3—H3	0.833 (10)	C2—C3	1.394 (3)
O4—C9	1.362 (2)	C3—C4	1.392 (3)
O4—H4	0.835 (10)	C4—C5	1.381 (3)
O5—C10	1.374 (2)	C4—H4A	0.9500
O5—H5	0.831 (10)	C5—C6	1.399 (3)
O6—C11	1.373 (2)	C5—H5A	0.9500
O6—H6	0.835 (10)	C6—C7	1.445 (3)
O7—C17	1.360 (2)	C7—H7A	0.9500
O7—H7	0.830 (10)	C9—C10	1.394 (3)
O8—C18	1.369 (2)	C9—C14	1.406 (3)
O8—H8	0.837 (10)	C10—C11	1.389 (3)
O9—C19	1.367 (2)	C11—C12	1.385 (3)
O9—H9	0.840 (10)	C12—C13	1.386 (3)
N1—C7	1.288 (3)	C12—H12	0.9500
N1—N2	1.385 (2)	C13—C14	1.398 (3)
N2—C8	1.344 (3)	C13—H13	0.9500
N2—H21	0.860 (10)	C14—C15	1.447 (3)
N3—C8	1.320 (3)	C15—H15	0.9500
N3—H31	0.851 (10)	C17—C18	1.398 (3)
N3—H32	0.853 (10)	C17—C22	1.402 (3)
N4—C15	1.285 (3)	C18—C19	1.390 (3)
N4—N5	1.385 (2)	C19—C20	1.394 (3)
N5—C16	1.349 (3)	C20—C21	1.380 (3)
N5—H51	0.865 (10)	C20—H20	0.9500
N6—C16	1.323 (3)	C21—C22	1.399 (3)
N6—H61	0.858 (10)	C21—H21a	0.9500
N6—H62	0.849 (10)	C22—C23	1.454 (3)
N7—C23	1.288 (3)	C23—H23	0.9500
			
C1—O1—H1	108.4 (19)	N3—C8—N2	118.19 (19)
C2—O2—H2	110 (2)	N3—C8—S1	123.33 (16)
C3—O3—H3	108.4 (19)	N2—C8—S1	118.47 (16)
C9—O4—H4	112 (2)	O4—C9—C10	117.13 (19)
C10—O5—H5	104 (2)	O4—C9—C14	122.83 (19)
C11—O6—H6	107.5 (19)	C10—C9—C14	120.01 (19)
C17—O7—H7	106 (2)	O5—C10—C11	122.75 (19)
C18—O8—H8	109 (2)	O5—C10—C9	117.48 (19)
C19—O9—H9	108.1 (18)	C11—C10—C9	119.76 (19)
C7—N1—N2	114.97 (17)	O6—C11—C12	123.38 (19)
C8—N2—N1	120.71 (17)	O6—C11—C10	115.75 (19)
C8—N2—H21	119.4 (17)	C12—C11—C10	120.87 (19)
N1—N2—H21	119.2 (17)	C13—C12—C11	119.3 (2)
C8—N3—H31	119.2 (19)	C13—C12—H12	120.3
C8—N3—H32	121.1 (16)	C11—C12—H12	120.3
H31—N3—H32	119 (2)	C12—C13—C14	121.2 (2)
C15—N4—N5	116.06 (18)	C12—C13—H13	119.4
C16—N5—N4	119.95 (18)	C14—C13—H13	119.4
C16—N5—H51	122.7 (19)	C13—C14—C9	118.70 (19)
N4—N5—H51	116.4 (19)	C13—C14—C15	119.20 (19)
C16—N6—H61	121.8 (17)	C9—C14—C15	122.01 (19)
C16—N6—H62	119.6 (17)	N4—C15—C14	122.3 (2)
H61—N6—H62	118 (2)	N4—C15—H15	118.9
C23—N7—N8	114.78 (18)	C14—C15—H15	118.9
C24—N8—N7	120.06 (18)	N6—C16—N5	117.59 (19)
C24—N8—H81	118.3 (16)	N6—C16—S2	123.38 (16)
N7—N8—H81	121.4 (16)	N5—C16—S2	119.03 (16)
C24—N9—H91	120.2 (17)	O7—C17—C18	117.13 (19)
C24—N9—H92	118.8 (18)	O7—C17—C22	123.12 (19)
H91—N9—H92	121 (2)	C18—C17—C22	119.74 (19)
O1—C1—C2	118.45 (19)	O8—C18—C19	122.09 (19)
O1—C1—C6	121.89 (18)	O8—C18—C17	117.80 (18)
C2—C1—C6	119.65 (19)	C19—C18—C17	120.11 (19)
O2—C2—C1	119.04 (18)	O9—C19—C18	115.08 (19)
O2—C2—C3	120.19 (18)	O9—C19—C20	124.29 (19)
C1—C2—C3	120.69 (19)	C18—C19—C20	120.6 (2)
O3—C3—C4	123.43 (18)	C21—C20—C19	118.9 (2)
O3—C3—C2	116.36 (18)	C21—C20—H20	120.5
C4—C3—C2	120.20 (19)	C19—C20—H20	120.5
C5—C4—C3	119.01 (19)	C20—C21—C22	121.8 (2)
C5—C4—H4A	120.5	C20—C21—H21a	119.1
C3—C4—H4A	120.5	C22—C21—H21a	119.1
C4—C5—C6	121.78 (19)	C21—C22—C17	118.79 (19)
C4—C5—H5A	119.1	C21—C22—C23	118.05 (19)
C6—C5—H5A	119.1	C17—C22—C23	123.11 (19)
C5—C6—C1	118.54 (19)	N7—C23—C22	122.12 (19)
C5—C6—C7	118.53 (19)	N7—C23—H23	118.9
C1—C6—C7	122.85 (19)	C22—C23—H23	118.9
N1—C7—C6	122.49 (19)	N9—C24—N8	118.0 (2)
N1—C7—H7A	118.8	N9—C24—S3	123.12 (17)
C6—C7—H7A	118.8	N8—C24—S3	118.90 (16)
			
C7—N1—N2—C8	−173.3 (2)	C11—C12—C13—C14	1.5 (3)
C15—N4—N5—C16	179.99 (19)	C12—C13—C14—C9	1.6 (3)
C23—N7—N8—C24	174.1 (2)	C12—C13—C14—C15	−175.1 (2)
O1—C1—C2—O2	3.8 (3)	O4—C9—C14—C13	177.55 (19)
C6—C1—C2—O2	−176.09 (19)	C10—C9—C14—C13	−4.6 (3)
O1—C1—C2—C3	−179.37 (19)	O4—C9—C14—C15	−5.9 (3)
C6—C1—C2—C3	0.8 (3)	C10—C9—C14—C15	172.0 (2)
O2—C2—C3—O3	−0.7 (3)	N5—N4—C15—C14	−176.27 (18)
C1—C2—C3—O3	−177.53 (19)	C13—C14—C15—N4	179.4 (2)
O2—C2—C3—C4	179.20 (19)	C9—C14—C15—N4	2.9 (3)
C1—C2—C3—C4	2.4 (3)	N4—N5—C16—N6	1.8 (3)
O3—C3—C4—C5	176.8 (2)	N4—N5—C16—S2	−178.53 (15)
C2—C3—C4—C5	−3.1 (3)	O7—C17—C18—O8	−1.8 (3)
C3—C4—C5—C6	0.7 (3)	C22—C17—C18—O8	178.62 (19)
C4—C5—C6—C1	2.4 (3)	O7—C17—C18—C19	178.8 (2)
C4—C5—C6—C7	−174.4 (2)	C22—C17—C18—C19	−0.8 (3)
O1—C1—C6—C5	177.06 (19)	O8—C18—C19—O9	−0.4 (3)
C2—C1—C6—C5	−3.1 (3)	C17—C18—C19—O9	178.91 (19)
O1—C1—C6—C7	−6.3 (3)	O8—C18—C19—C20	179.7 (2)
C2—C1—C6—C7	173.6 (2)	C17—C18—C19—C20	−1.0 (3)
N2—N1—C7—C6	−175.78 (18)	O9—C19—C20—C21	−178.4 (2)
C5—C6—C7—N1	176.6 (2)	C18—C19—C20—C21	1.5 (3)
C1—C6—C7—N1	−0.1 (3)	C19—C20—C21—C22	−0.3 (3)
N1—N2—C8—N3	1.5 (3)	C20—C21—C22—C17	−1.4 (3)
N1—N2—C8—S1	−179.28 (15)	C20—C21—C22—C23	176.0 (2)
O4—C9—C10—O5	3.3 (3)	O7—C17—C22—C21	−177.6 (2)
C14—C9—C10—O5	−174.69 (19)	C18—C17—C22—C21	1.9 (3)
O4—C9—C10—C11	−177.45 (18)	O7—C17—C22—C23	5.2 (4)
C14—C9—C10—C11	4.5 (3)	C18—C17—C22—C23	−175.3 (2)
O5—C10—C11—O6	−2.0 (3)	N8—N7—C23—C22	177.28 (19)
C9—C10—C11—O6	178.77 (19)	C21—C22—C23—N7	−177.5 (2)
O5—C10—C11—C12	177.7 (2)	C17—C22—C23—N7	−0.3 (4)
C9—C10—C11—C12	−1.5 (3)	N7—N8—C24—N9	0.3 (3)
O6—C11—C12—C13	178.2 (2)	N7—N8—C24—S3	−179.20 (16)
C10—C11—C12—C13	−1.6 (3)		

### Hydrogen-bond geometry (Å, °)

**Table:**

*D*—H···*A*	*D*—H	H···*A*	*D*···*A*	*D*—H···*A*
O1—H1···N1	0.84 (1)	1.94 (2)	2.682 (2)	147 (3)
O2—H2···O3^i^	0.83 (1)	2.17 (2)	2.773 (2)	129 (3)
O3—H3···S1^ii^	0.83 (1)	2.36 (1)	3.184 (2)	173 (3)
O4—H4···N4	0.84 (1)	1.96 (2)	2.666 (2)	141 (3)
O5—H5···O6	0.83 (1)	2.24 (3)	2.730 (2)	118 (3)
O6—H6···S2^ii^	0.84 (1)	2.45 (1)	3.276 (2)	172 (2)
O7—H7···N7	0.83 (1)	1.97 (2)	2.710 (2)	148 (3)
O8—H8···O9	0.84 (1)	2.24 (3)	2.697 (2)	114 (2)
O9—H9···S3^iii^	0.84 (1)	2.40 (1)	3.237 (2)	174 (2)
N3—H31···O2^iv^	0.85 (1)	2.09 (1)	2.902 (2)	159 (3)
N6—H61···O8^v^	0.86 (1)	2.01 (1)	2.847 (2)	164 (2)
N9—H91···O5^vi^	0.86 (1)	2.11 (2)	2.896 (2)	153 (2)

Symmetry codes: (i) −*x*, −*y*+1, −*z*; (ii) *x*, *y*, *z*−1; (iii) *x*, *y*, *z*+1; (iv) −*x*, −*y*+1, −*z*+1; (v) *x*, *y*−1, *z*; (vi) *x*, *y*+1, *z*.
